# The London Hospital and the National Insurance Act

**Published:** 1913-06-14

**Authors:** 


### THE LONDON HOSPITAL AND THE NATIONAL INSURANCE ACT.

We are indebted to the authorities of the London Hospital for some interesting particulars of the work done there during the past quarter in connection with the Insurance Act. We hope that the authorities and secretaries of other hospitals throughout the country will kindly send us particulars of anything that they are doing in the matters referred to and of any steps or developments at their institutions arising out of the Insurance Act, or otherwise. We take the following information, edited up to date, from the quarterly report of the governors of the London Hospital:—

### An Important Modification in Practice.

As regards the Insurance Act, though protest was made before the passing of the measure against the manner in which the hospitals were entirely ignored, on the Bill becoming law your committee used every endeavour to co-operate with the working of the Act in order to render it as effective as possible. It will be remembered that it was decided to refer to panel doctors insured persons who applied to the hospital, but who were suffering from such minor complaints as could be treated just as competently by a panel doctor as by the hospital doctors. Recently, however, the committee have found it necessary to modify this regulation in favour of patients suffering from minor accidents. Difficulties arose owing to individuals who, though employed in factories near the hospital, lived at some distance from the hospital, and, accordingly, their panel doctors were not available in cases of emergency. For instance, it was found to be a hardship to refer an individual suffering perhaps from a severely cut hand to his panel doctor if his panel doctor resided some long distance away from the hospital. Your committee therefore decided that such accident cases should be treated at the hospital.

### The Hospital as a Recognised Tuberculosis Centre.

Negotiations are pending with the London County Council with regard to this hospital being recognised as a tuberculosis centre for the treatment of insured persons suffering from tuberculosis. No definite arrangements have so far been made, but the London County Council is actively engaged in formulating a comprehensive scheme which will effectively deal with the problem. In this scheme it is understood that the County Council is willing, and, in fact, is wishful to make use of existing institutions. It is hardly necessary to point out to the governors that at this hospital there is an eminent staff of specialists and bacteriologists. Your committee have expressed their intention to co-operate in every way possible with the London County Council in the scheme which it is hoped will ultimately wipe out the scourge of tuberculosis.

### Sickness Benefit under the Act.

As the members of the Court are doubtless aware the sickness benefit under the Act of 10s. to a male and 7s. 6d. to a female is not payable to the individual while an inmate of a hospital or kindred voluntary institution. If the individual, however, has any dependants he or she may nominate such dependants to receive the sickness benefit during the period of stay in hospital. In the event, however, of insured persons having no dependants, then, according to the Act, the friendly societies may assign such sickness benefit to the hospital. A record has been kept of insured persons treated in the hospital who have no dependants, and the various friendly societies have been approached with a view of getting them to agree to transfer in such cases the sickness benefit to the hospital. If this principle were generally established the amount which the hospital would receive would be considerable, since from one large society alone it is estimated that a sum of about £400 per annum would probably be transferred to the hospital. Difficulties have arisen, however, owing to the fact that there is no obligation expressed in the Act to transfer sickness benefit to the hospital in the cases of in-patients who have no dependants. Representations have, however, been made to the Insurance Commissioners, and it is hoped that pressure will be brought upon the approved societies so that the handing over to the hospital of the sickness benefit of insured persons who have no dependants may become obligatory. At present, however, the approved society refuses because as the Act at present stands there is no compulsion about it. These approved societies are in competition, and any society which consented to hand over sickness benefit to a hospital would be at a disadvantage with every other society which retained these benefits and expends them on the patient when he leaves the hospital.

### Maternity Benefit and Hospital Patients.

As regards maternity benefit, the wife of an insured person under the Act receives 30s. for her confinement. Under these circumstances it seemed irrational that the hospital should attend a woman for her confinement gratuitously. The Court are doubtless aware that the hospital doctors and midwives attend patients within a mile radius of the hospital for confinement. A certain number are also admitted to the nine Marie Celeste Maternity Beds. The committee decided to demand a contribution of 10s. per patient, though the secretary was given a certain amount of latitude to make a reduction if the circumstances of any particular case so demanded.

### The Attendance of Sick Nurses.

With regard to the treatment of our nurses when sick, it has been decided, after negotiations with the Insurance Committee for the County of London, to arrange for one of our doctors under an impersonal title to join what is known as the "starred panel"—that is to say, the doctor will be available solely for the treatment of our nurses and other resident employees. According to this plan the sum of 9s. per individual per annum will be received from the Insurance Committee, the total amount probably being £315 per annum. This will be a sum set off—though, of course, some remuneration will have to be paid to our doctor—to the £436 which our nurses cost the hospital by way of insurance under the Act.

### L.C.C. Aural, Ophthalmic, Skin, and Dental Cases in Children.

Your committee decided to renew for a further period up to July 31, 1914, the agreement with the London County Council by which 3,000 aural, 3,500 ophthalmic, and 350 skin cases are treated per annum. Negotiations have also been pending with the County Council for the treatment of school children in our dental department. Your committee have now agreed to treat 1,540 school children, and the scheme only awaits final arrangements. It may be remembered that on a previous occasion the committee refused to enter into an agreement for the treatment in our dental department of school children. The recent enlargement, however, of our dental department has now made it possible to agree to the request of the County Council.

### The Results of the Quinquennial Appeal.

As regards the quinquennial appeal, unfortunately this has so far not been a success, which we anticipated and hoped. Up to May 31 the sum of £56,448 has been received. This includes an anonymous gift of £10,000 of bearer bonds of good security. The appeal this year has been most systematically made, but the results have been disappointing. A full-page advertisement was inserted in the leading daily and evening papers. Mr. Filson Young, Mr. Pett Ridge, and Mr. M. Piggott also most kindly wrote articles in support of the advertisement. The amount, however, received from the full-page advertisement, which was inserted in altogether fourteen newspapers, was only £919 16s. 2d. This result would seem to indicate that mere advertisement in daily papers on behalf of a charity was certainly not profitable. The Lord Mayor most kindly issued on behalf of the hospital a personal appeal, which has up to date brought in about £2,000. He also very kindly agreed to hold a banquet on behalf of the hospital at the Mansion House. This, unfortunately, has had to be postponed. An appeal to banks has so far realised £5,227 5s. It is also intended to organise an appeal on the Stock Exchange and on Lloyd's. It is satisfactory to learn, however, that this appeal is, so far, as successful as that of five years ago.

### A Fund for Free Radium Supplies.

It is with feelings of gratitude that an announcement is made of the gift of £1,000 from Mrs. Stewart MacKenzie for the purchase of radium. The possibilities of radium for curative purposes have now been satisfactorily demonstrated, and the hospital has for some time past laboured under great disabilities owing to having insufficient quantity. The amount which this munificent gift will purchase, though by no means bringing the quantity of radium in the possession of the hospital up to a large amount, will enable more patients to be treated with a far greater degree of efficiency.

